# Development of a Screening Tool for Sleep Disordered Breathing in Children Using the Phone Oximeter™

**DOI:** 10.1371/journal.pone.0112959

**Published:** 2014-11-17

**Authors:** Ainara Garde, Parastoo Dehkordi, Walter Karlen, David Wensley, J. Mark Ansermino, Guy A. Dumont

**Affiliations:** 1 Electrical and Computer Engineering in Medicine Group, The University of British Columbia and BC Children's Hospital, Vancouver, British Columbia, Canada; 2 Anesthesiology, Pharmacology and Therapeutics, The University of British Columbia and BC Children's Hospital, Vancouver, British Columbia, Canada; 3 Division of Critical Care, University of British Columbia and BC Children's Hospital, Vancouver, British Columbia, Canada; Laboratorio de Neurociencias Moleculares e Integrativas. Escuela de Medicina, División Ciencias de la Salud. Universidad Anáhuac Mayab. Mérida, Yucatán. México

## Abstract

**Background:**

Sleep disordered breathing (SDB) can lead to daytime sleepiness, growth failure and developmental delay in children. Polysomnography (PSG), the gold standard to diagnose SDB, is a highly resource-intensive test, confined to the sleep laboratory.

**Aim:**

To combine the blood oxygen saturation (SpO_2_) characterization and cardiac modulation, quantified by pulse rate variability (PRV), to identify children with SDB using the Phone Oximeter, a device integrating a pulse oximeter with a smartphone.

**Methods:**

Following ethics approval and informed consent, 160 children referred to British Columbia Children's Hospital for overnight PSG were recruited. A second pulse oximeter sensor applied to the finger adjacent to the one used for standard PSG was attached to the Phone Oximeter to record overnight pulse oximetry (SpO_2_ and photoplethysmogram (PPG)) alongside the PSG.

**Results:**

We studied 146 children through the analysis of the SpO_2_ pattern, and PRV as an estimate of heart rate variability calculated from the PPG. SpO_2_ variability and SpO_2_ spectral power at low frequency, was significantly higher in children with SDB due to the modulation provoked by airway obstruction during sleep (*p*-value 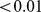). PRV analysis reflected a significant augmentation of sympathetic activity provoked by intermittent hypoxia in SDB children. A linear classifier was trained with the most discriminating features to identify children with SDB. The classifier was validated with internal and external cross-validation, providing a high negative predictive value (92.6%) and a good balance between sensitivity (88.4%) and specificity (83.6%). Combining SpO_2_ and PRV analysis improved the classification performance, providing an area under the receiver operating characteristic curve of 88%, beyond the 82% achieved using SpO_2_ analysis alone.

**Conclusions:**

These results demonstrate that the implementation of this algorithm in the Phone Oximeter will provide an improved portable, at-home screening tool, with the capability of monitoring patients over multiple nights.

## Introduction

Sleep disordered breathing (SDB) describes a family of disorders characterized by frequent partial or complete cessations of breathing during sleep. SDB is a common and highly prevalent condition in children (2% among children [1], [2] and 2.5%-6% among adolescents [3]) that can cause severe complications if left untreated. Symptoms include snoring, disturbed sleep, daytime sleepiness and neurobehavioural problems [4],[5]. SDB includes obstructive sleep apnea (OSA) syndrome, central sleep apnea syndrome, Cheyne-Stokes respiration, and alveolar hypoventilation syndrome [6]. OSA is the most common type of SDB in children and is characterized by repeated obstruction of breathing during sleep, which results in oxyhemoglobin desaturation, hypercapnia and repeated arousals. Complications due to recurrent hypoxia-reoxygenation episodes during the night, include neurocognitive impairment, behavioural problems, failure to thrive, and cor pulmonale, particularly in severe cases [7],[8]. Thus, SDB poses a serious threat to the healthy growth and development of many children.

Polysomnography (PSG), the gold standard to diagnose SDB, is the most commonly used diagnostic technique shown to quantify the ventilatory and sleep abnormalities associated with SDB. This nocturnal study is highly resource-intensive [9],[10] and requires a specialized sleep laboratory, expensive equipment and an overnight stay in the facility [6], confining PSG monitoring to centralized specialist facilities. For example, in British Columbia all PSG studies in children are performed at the British Columbia Children's Hospital (BCCH) in Vancouver. This greatly limits access, especially for those who live in remote locations. The capacity to perform PSG at BCCH is limited to fewer than 250 cases per year, resulting in a waitlist of six months. In recently developed clinical practice guidelines for the diagnosis and management of SDB in children and adolescents [4], the American Academy of Pediatrics concludes that all children/adolescents should be screened for snoring and OSA symptoms (defined in the guidelines [4],[7]) and PSG should be performed only in those with regular snoring and signs of OSA.

The high cost (approximately $800 per night in direct health care costs at BCCH) [11] and limited access of PSG have generated a great interest in alternative techniques to simplify the standard procedure. Already part of the standard PSG, pulse oximetry is a simple non-invasive method of measuring blood oxygen saturation (SpO_2_) and recording blood volume changes in tissue using the photoplethysmographic signal (PPG). Numerous groups have studied the use of overnight oximetry as a potential standalone method to diagnose SDB. Nixon et al. developed a severity scoring system using overnight oximetry and validated the score as a tool to prioritize adenotonsillectomy surgeries [12],[13]. Álvarez et al. demonstrated that the characterization of overnight oximetry provided significant information to identify adults [14],[15] with significant OSA. Both studies focused on SpO_2_ alone; however, there are some SDB events that occur in the absence of SpO_2_ desaturation [16]. It has been reported that SDB affects the normal variation of heart rate [17],[18], suggesting that combining SpO_2_ and Heart Rate Variability (HRV) analysis might provide a more robust SDB detector. Based on this concept, Heneghan et al. proposed a portable, automated OSA assessment tool with a Holter-Oximeter [19], [20].

**Figure 1:**
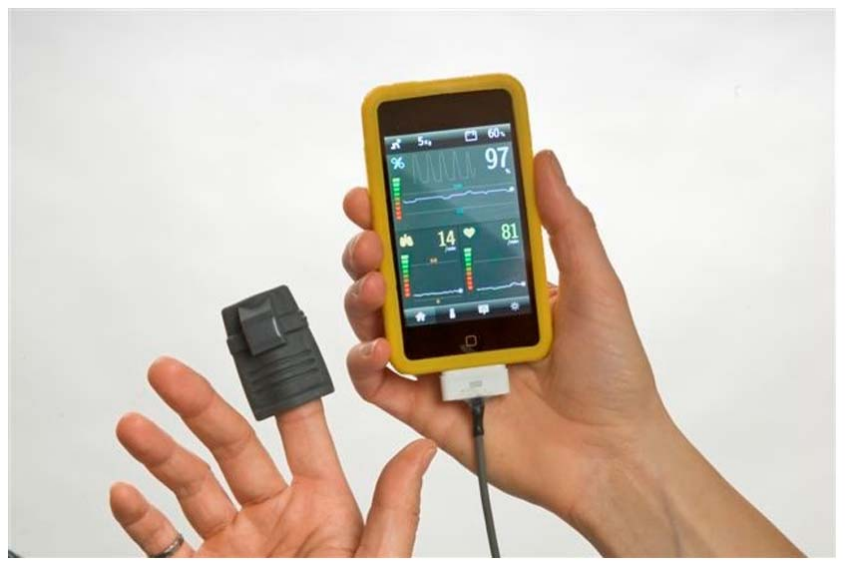
The Phone Oximeter. A mobile device that integrates a pulse oximeter with a smartphone.

The original Phone Oximeter (Figure 1) is a mobile device that integrates a commercially available and Federal Drug Administration (FDA) approved microcontroller-based pulse oximeter (Masimo Set uSpO_2_ Pulse Oximetry Cable) with a mobile smartphone [21]. The Phone Oximeter enables the acquisition, monitoring and analysis of vital signs and intuitive display of information to health care providers. A low cost version of the Phone Oximeter that does not require an intermediate microcontroller was recently developed. This prototype interfaces the sensor directly with the phone via the audio jack, reducing the total cost of the Phone Oximeter to only that of the finger probe [22],[23]. In our previous research, we showed that the characterization of overnight SpO_2_ pattern, measured by the Phone Oximeter, successfully identifies children with significant SDB [24]. We also investigated the influence of SpO_2_ resolution (0.1%, 1%) on the SpO_2_ pattern characterization and demonstrated that it has a great influence in regularity measurements and therefore should be considered when studying SDB [25]. In addition, we calculated Pulse Rate Variability (PRV) from the Phone Oximeter's PPG, and compared it with HRV computed from simultaneous electrocardiogram (ECG) [26]. In the time domain, PRV provided accurate estimates of HRV, while some differences were found in the frequency domain. Gil et al. also showed that during non-stationary conditions there are some small differences between HRV and PRV, mainly in the respiratory band, which were related to the pulse transit time variability [27]. However, they also concluded that these differences are sufficiently small to suggest the use of PRV as an alternative measure of HRV. We also conducted an additional investigation of the effects of SDB on PRV during different sleep stages and concluded that the modulation of PRV might be helpful in improving the assessment of SDB in children [28]. Therefore, the purpose of this study is to combine both SpO_2_ pattern characterization and PRV analysis to identify children with significant SDB, using the Phone Oximeter. We evaluate the Phone Oximeter's potential as a stand-alone SDB screening tool to identify children who should undergo a complete PSG study, with the eventual goal of reducing costs and hospital waitlists.

## Material and Methods

### 2.1 Dataset

#### 2.1.1 Ethics statement

All subjects were recruited according to a protocol approved by the University of British Columbia and Children's and Women's Health Centre of British Columbia Research Ethics Board (H11-01769). Parental/guardian written informed consent was obtained for all subjects, and written assent was obtained for all subjects over the age of 11 years.

#### 2.1.2 Data acquisition

One hundred and sixty children with signs of sleep apnea (such as snoring, daytime sleepiness, behavioural problems or clinically large tonsils) referred to BCCH for PSG recording were recruited to and participated in this study. Children with cardiac arrhythmia or abnormal hemoglobin were excluded. In addition, fourteen children were excluded from the study because the total duration of the sleep time or the collected signals from the PSG or the Phone Oximeter (PPG and SpO_2_) were shorter than 3 hours.

The data acquisition was carried out in the sleep unit. Standard PSG was recorded using the Embla Sandman S4500, specifically designed to meet the American Academy of Sleep Medicine (AASM) accreditation requirements. The PSG included the overnight measurement of ECG, electroencephalogram (EEG), SpO_2_, chest and abdominal movement, nasal and oral airflow, and video recordings. The pulse oximeter sensor of the Phone Oximeter was applied to the finger adjacent to the one used during standard PSG. The SpO_2_ (0.1% resolution) and PPG signals, recorded by the Phone Oximeter, were sampled at 1 Hz and 62.5 Hz, respectively.

A sleep technician visually scored the PSG in 30-second epochs according to AASM 2007 standard criteria [29]. Hypnograms were differentiated into stage 1, stage 2, stage 3 (non-REM) and rapid eye movement (REM) sleep. According to the standard criteria, obstructive apneas were defined as complete cessation of airflow in the presence of respiratory effort lasting 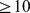 seconds. Hypopneas were defined as a 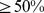 airflow reduction relative to the 2 preceding breaths. Blood oxygen desaturations were defined as a 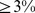 decrease in arterial oxygen saturation. When respiratory effort partially or totally ceased, apneas were scored as mixed or central sleep apnea, respectively. The number of apneas/hypopneas was counted hourly to compute the average apneas/hypopnea index (AHI), which was specified also for REM and non-REM (NREM) sleep stages. The total bed time (TBT), total sleep time (TST) and the percentage of time spent in the different sleep stages were also analyzed (Table 1). Pulse oximetry data acquired with the Phone Oximeter and the reference AHI is be publicly available online via doi:10.6084/m9.figshare.1209662.

**Table 1:** Demographic and PSG information in the study group (mean ± standard deviation).

Dataset	SDB	NonSDB
Number(F, M)	56 (18, 38)	90 (41, 49)
Age (y)	8.8 ± 4.6	9.3 ± 4
AHI (apnea hypoapnea/hour)	19.7 ± 19.5**	1.4 ± 1.1
AHI in REM†	34.8 ± 27.8**	4.4 ± 5.1
AHI in NREM	15.8 ± 22.8**	0.76 ± 0.96
Lowest SpO_2_ (%)	82.3 ± 15.0*	90.1 ± 3.5
BMI (kg/m2)	23.2 ± 8.3*	19.6 ± 6.6
Sleep efficiency (%)	75.1 ± 16.2	76.6 ± 15.3
TST (min)	362.1 ± 82.6	368 ± 73.8
TBT (min)	479.9 ± 40	481.4 ± 24.1
Stage 1 (%)	6.5 ± 5.9	5 ± 3.2
Stage 2 (%)	56.9 ± 12.8	59.4 ± 10.4
Stage 3 (%)	15.3 ± 9.6	17.3 ± 9.1
REM (%)	20.2 ± 8	18.2 ± 6.1
Awakenings	21.2 ± 10.6	18.6 ± 9.3
Respiratory arousals	13.6 ± 13.9**	1 ± 0.9

*p-value <0.001, **p-value <0.0001 comparing SDB and NonSDB.

† *p*-value <0.001 comparing AHI in REM and NREM sleep stages.

The current clinical protocol at BCCH recommends children with an AHI 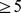 undergo treatment for SDB. This study therefore used an AHI 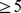 as a positive indication of SDB. The current treatment for SDB at BCCH consists of continuous or bi-level positive airway pressure (CPAP or BiPAP) or surgical adenotonsillectomy. Adenotonsillectomy being the most common treatment for pediatric SDB [12],[30].

### 2.2 Characterization

The proposed algorithm characterizes both the SpO_2_ pattern [24] and PRV [28], in the time and frequency domains, using a 2-minute sliding window, with 1-minute overlap. This characterization was performed offline in Matlab (Mathworks Inc, Natick, USA).

#### 2.2.1 SpO_2_ pattern

All SpO_2_ values below 50% and above 100%, and SpO_2_ changes between consecutive sampling intervals greater than 4%, were considered as artifacts and eliminated prior to further analysis. The SpO_2_ signal analysis was focused on characterizing modulation generated by the desaturations resulting from SDB.

**Time domain features:** A number of time domain statistics such as: mean, median, standard deviation and interquartile range of the SpO_2_ as well as indices including the number of desaturations from baseline below 2% (n2%), the cumulative time spent below 92% (t92%) and the Δ index (variability measure) were calculated for each time window. In addition, to evaluate the complexity of the SpO_2_ pattern, nonlinear measures such as sample entropy (SampEn), approximate entropy (ApEn) and central tendency measure (CTM) [31], [32] were calculated.

**Spectral domain features:** The SpO_2_ signal was characterized in the spectral domain using power spectral density (PSD). To provide better frequency resolution, a parametric PSD was performed approximating the SpO_2_ signal through an autoregressive model using:
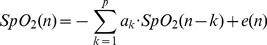
(1)


where *e(n)* denotes zero-mean white noise with variance 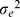, 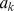 the autoregressive coefficients and 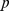 the model order. Once the autoregressive coefficients and the variance was estimated, the PSD of an autoregressive model was computed by:
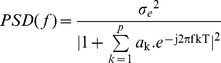
(2)


With 1/T as the sampling frequency.

**Figure 2:**
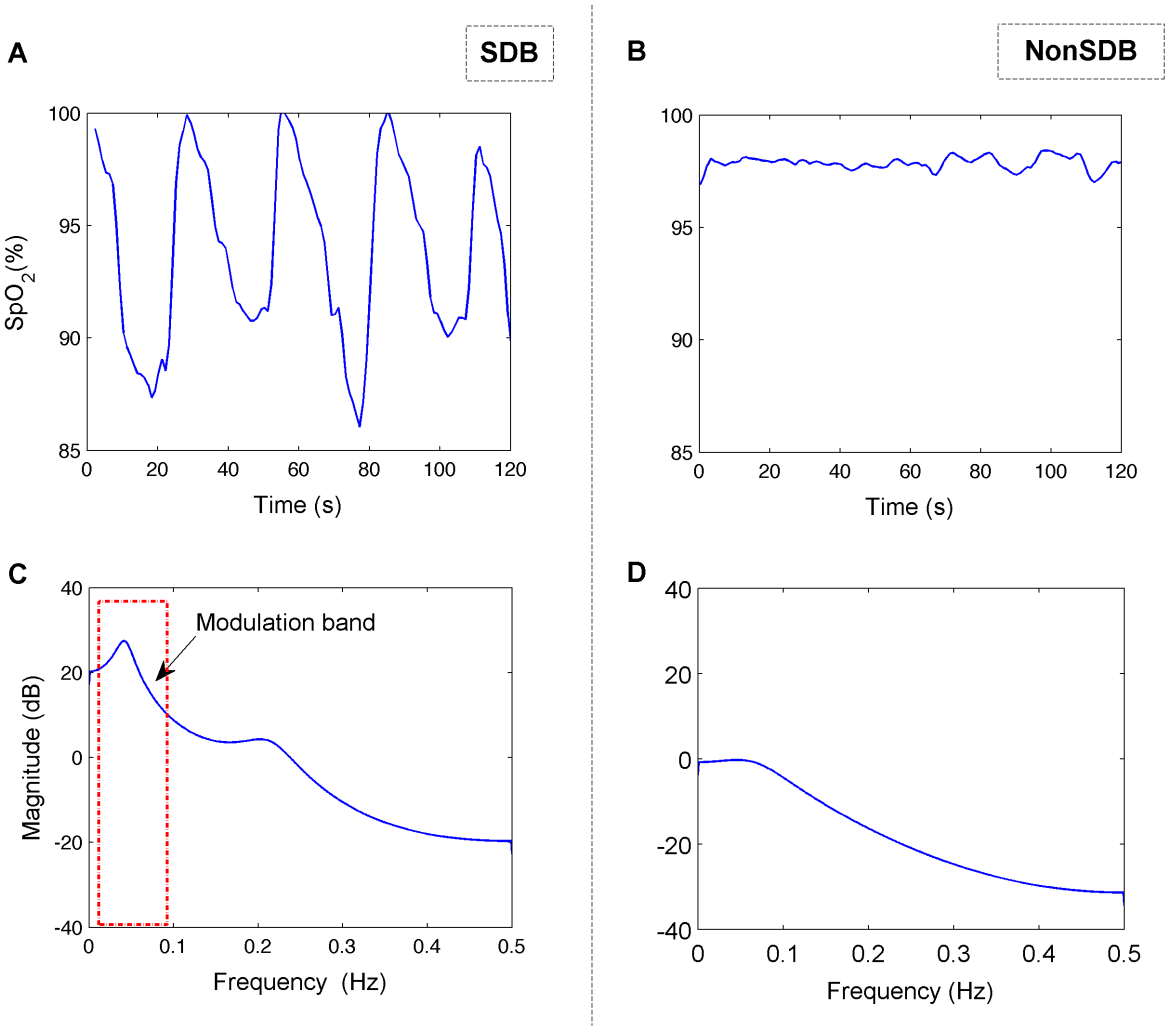
Power spectral density applied to a 2-minute SpO_2_ signal of (A) a child with, and (B) without SDB. The SDB child shows a clear modulation frequency peak, whereas the NonSDB child illustrates no clear modulation peak.

**Figure 3:**
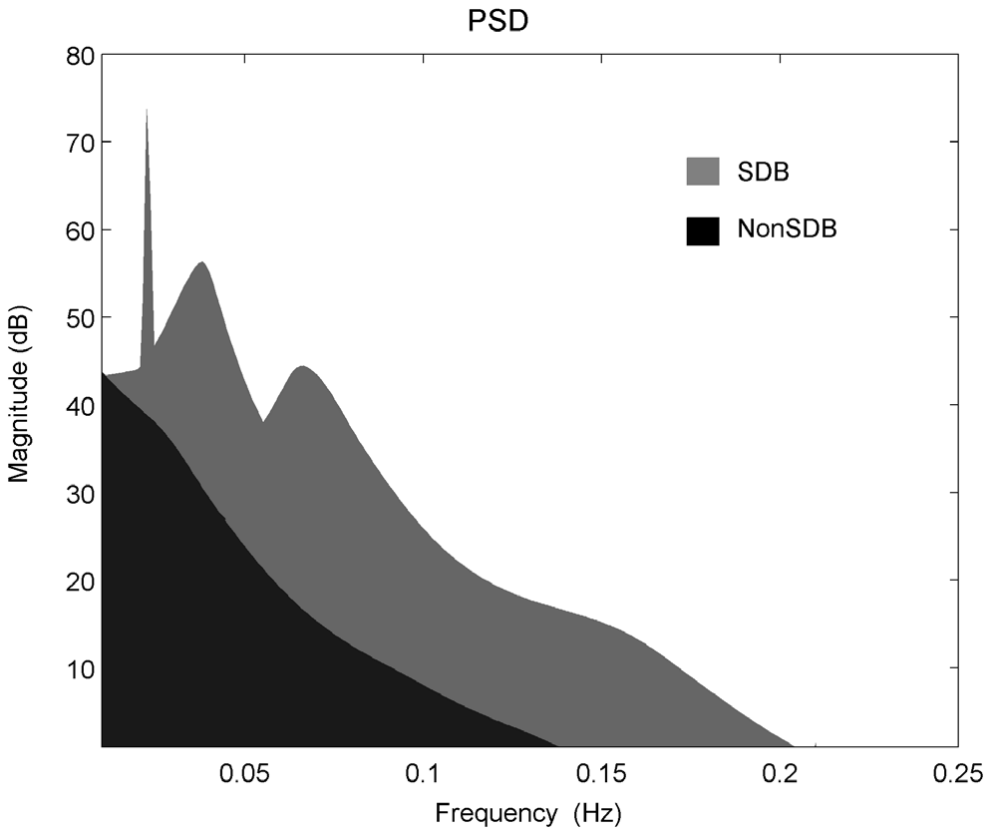
PSD applied to the SpO_2_ of whole study population. The mean PSD (average of the PSDs obtained for each time window overnight) for each SDB subject is represented in light grey, and NonSDB is represented in dark grey.

**Figure 4:**
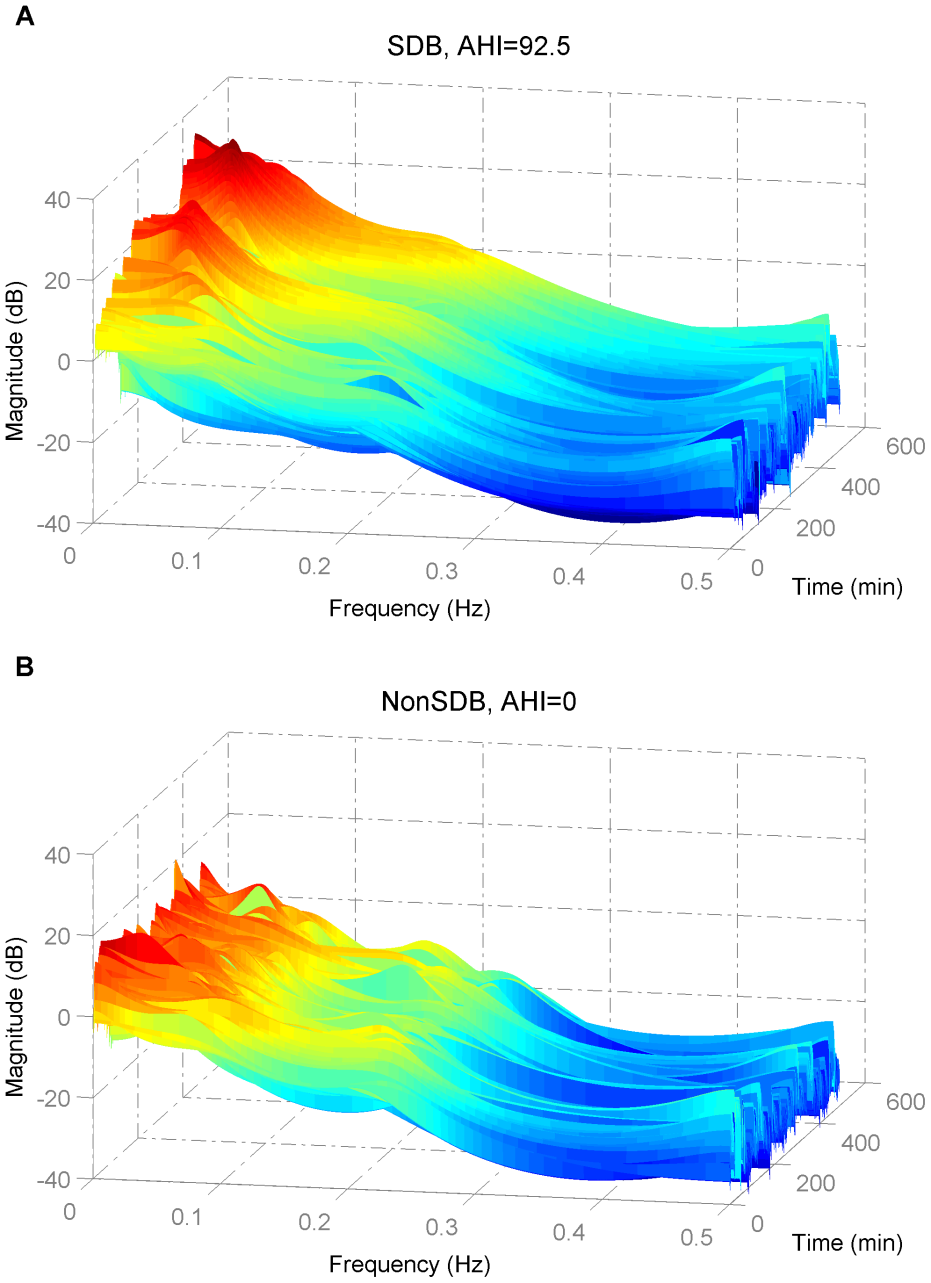
Time-varying power spectral density applied to an overnight SpO_2_ signal of (A) a child with and (B) without SDB. The SDB child shows a clear modulation frequency peak and higher energy around this peak compared to the NonSDB child.

The selection of model order is a trade-off between the frequency resolution and the presence of spurious peaks. The optimum model order was selected according to the minimum description length criterion from Rissanen [33]. Using the 2-minute sliding window, the SpO_2_ signal was divided into small segments that can be assumed to be stationary and therefore, permit computation of PSD (see Figure 2). The sleep apnea events happen in a pseudo periodic pattern, which modulates the SpO_2_ signal and provokes a modulation frequency peak at very low frequency band (Figure 3). In addition, this time-varying spectral analysis permitted consideration of the SpO_2_ pattern changes in the frequency domain (see Figure 4). Three spectral parameters were extracted from the PSD: 1) the power (P) within the modulation band (which consists of a frequency interval of 0.02 Hz centered around the modulation frequency peak, tracked in the band from 0.005 to 0.1 Hz); 2) the ratio (R) between the power within the modulation band and total power; and 3) the Shannon entropy (SE) of the PSD.

#### 2.2.2 Pulse Rate Variability (PRV)

A baseline removal and smoothing Savitzky-Golay filter (order 3, frame size 11 samples) was applied to the PPG signal. A signal quality index, obtained by an adaptive version of the algorithm developed by Karlen et al. [34], was performed for automatic rejection of windows containing motion artifacts. In order to obtain the time series of pulse to pulse intervals (PPIs) for each window, the locations of the peak of pulses in each segment of PPG signal were detected by a simple zero-crossing algorithm. The intervals between successive peaks were subsequently computed. The PPIs shorter than 0.33 seconds or longer than 1.5 seconds were considered as artifacts and deleted from the time series. PRV was then obtained by converting each sequence of PPIs into an equivalent, uniformly spaced time series (sampling rate: 4 Hz), using a resampling method based on Berger et al. algorithm [35].

**Time domain features:** The mean of PPIs (representing RR, or the time interval between two consecutive R waves in the ECG), the standard deviation of PPIs (representing SDNN, or the standard deviation of the so-called normal-to-normal (NN) intervals), and the root mean square of the successive differences between adjacent PPIs (representing RMSSD, or the root mean square of the successive differences between adjacent NN intervals) were computed from each PPI time series.

**Spectral domain features:** The spectral PRV analysis was performed using a parametric PSD based on an autoregressive model of order 16. The power in each of the following frequency bands was computed by determining the area under the PSD curve bounded by the bandwidth: Very Low Frequency (VLF; 0.01–0.04 Hz), Low Frequency (LF; 0.04–0.15 Hz) and High Frequency (HF; 0.15–0.4 Hz). Normalized LF and HF powers were determined by dividing LF and HF powers by the total spectral power within the 0.04 and 0.4 Hz band. The ratio of the low-to-high frequency power (LF/HF ratio) was also computed.

### 2.3 Data Analysis

The described feature set characterizes the behaviour of the SpO_2_ signal and PRV for each 2-minute time window. However, to identify children with SDB, the statistical distribution of each time-varying parameter in the overnight recordings was evaluated through their means (M), medians (Me), standard deviations (S), and interquartile ranges (I) (see Table 2 for feature description).

**Table 2:** Parameter description and corresponding statistics.

Feature	Description	Statistics
	SpO_2_ pattern characterization	
**P**	Power modulation band	M_P, M_P, S_P, I_P
**R**	Power ratio (P/Total power)	M_R, Me_R, S_R, I_R
**SE**	Spectral Shannon entropy	M_SE, Me_SE, S_SE, I_SE
**Δ**	Delta index	M_Δ, Me_Δ, S_Δ, I_Δ
**iqr**	Inter quartile range	M_iqr, Me_iqr, S_iqr, I_iqr
**std**	Standard deviation	M_std, Me_std, S_std, I_std
**t94%**	Time spend below 94%	M_t94%, Me_t94%, S_t94%, I_t94%
**n2%**	Desaturations 2% below baseline	M_n2%, Me_n2%, S_n2%, I_n2%
**CTM**	Central tendency measure	M_CTM, Me_CTM, S_CTM, I_CTM
**ApEn**	Approximate entropy	M_ApEn, Me_ApEn, S_ApEn, I_ApEn
**SampEn**	Sample entropy	M_SampEn, Me_SampEn, S_SampEn, I_SampEn
	**PRV characterization**	
**LF**	Normalized power in low freq. band	M_LF, Me_LF, S_LF, I_LF
**HF**	Normalized power in high freq. band	M_HF, Me_HF, S_HF, I_HF
**LF/HF**	Ratio between LF/HF	M_LF/HF, Me_LF/HF, S_LF/HF, I_LF/HF
**RR**	Pulse to pulse interval (PPI)	M_RR, Me_RR, S_RR, I_RR
**SDNN**	Standard deviation of PPI	M_SDNN, Me_SDNN, S_SDNN, I_SDNN
**RMSSD**	Root mean square of standard deviation of PPI	M_RMSSD, Me_RMSSD, S_RMSSD, I_RMSSD

The normality of each feature was assessed using the Shapiro-Wilk test and visual inspection of the histograms and Q-Q plots. In order to evaluate the differences between SDB and NonSDB, two-sample t-tests for unequal variances were applied to the normally distributed features.

A logarithmic transformation was applied to non-normally distributed features to convert them into normally distributed variables. Two-sample t-tests for unequal variances were then calculated using log-transformed data. In addition, Mann-Whitney U tests were also computed using the original data. A probability of *p*-value 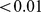 was considered significant and a Bonferroni correction was applied where appropriate.

### 2.4 Feature Selection and Classification

**Figure 5:**
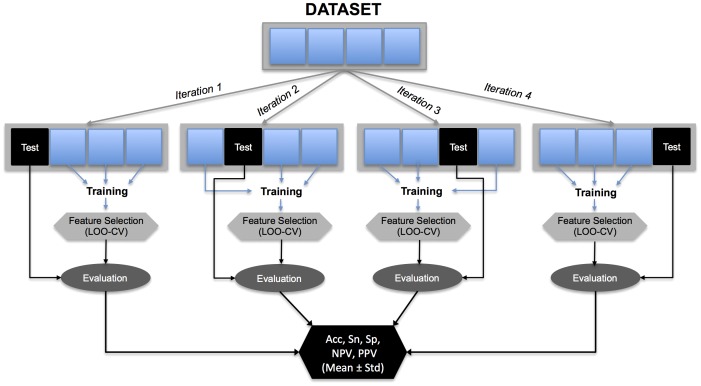
Diagram of the classification process with internal LOO and external 4-fold cross-validation (CV). The dataset was randomly divided into 4 non-overlapping subsets. 3 formed the training dataset and the remaining formed the test dataset. This process was repeated four times, until each subset was treated once as the test dataset. The most discriminant features classifying children with and without SD were selected using LOO-CV. This feature selection was then evaluated with the independent test dataset.

Linear discriminant analysis was performed to classify children with and without SDB. Based on the percentage of children with SDB in the dataset (39%), a prior probability of 0.4 was specified for the linear discriminant analysis. An external N-fold cross validation (N = 4) was used to estimate the performance of the linear discriminant. The dataset was randomly divided into N non-overlapping subsets. N-1 formed the training dataset (75% of the dataset, 110 children), and the remaining formed the test dataset (25% of the dataset, 36 children). Then, two tests were carried out. Firstly, the most discriminant features were selected using the training dataset. Secondly, the performance of this feature set was evaluated using the test dataset (Figure 5). This process was repeated N = 4 times, until each subset was treated once as the test dataset.

The features most effective for classifying children with and without SDB were selected using a feature selection algorithm, based on optimizing the area under the curve (AUC) of the Receiver Operating Characteristic (ROC) curve obtained with the linear classifier. An internal “leave-one-out” (LOO) cross-validation was applied to the feature selection to avoid a selection bias. Only the statistically significant parameters (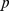-value 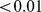) extracted from SpO_2_ and HRV analysis were applied to the feature selection process.

The results are represented in terms of accuracy, sensitivity, and specificity classifying children with significant SDB (AHI 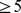). Positive and negative predictive values are also calculated to take into account the prevalence of SDB in this cohort. In addition, to quantify the benefits of combining SpO_2_ pattern characterization with PRV analysis, the same feature selection and classification was performed using only the features extracted from the SpO_2_ characterization.

## Results

### 3.1 Statistical Analysis

In total, we characterized the SpO_2_ pattern and PRV of 146 children (56 SDB, 90 NonSDB). The AHI was significantly higher during REM sleep stages, and as expected the Body Mass Index (BMI) was significantly higher in the SDB group [36] (see Table 1).

**Figure 6:**
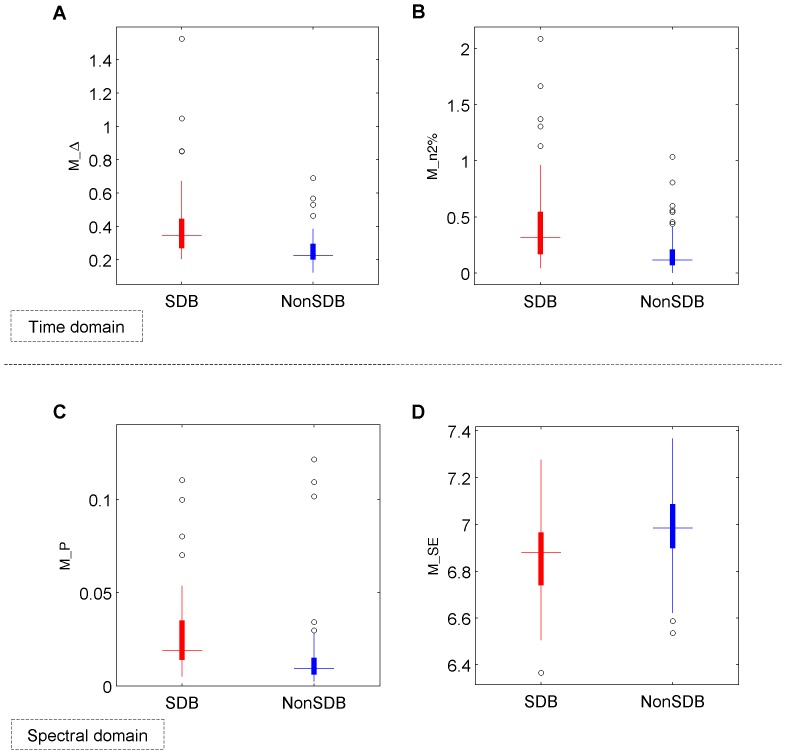
Distribution of SpO_2_ pattern characterization features. Boxplot of some features extracted from SpO_2_ pattern characterization such as (A) the mean of Δ (M_Δ), (B) the number of desaturations of 2% below baseline (M_n2%), (C) the spectral power in the modulation band (M_P), and (D) the spectral Shannon entropy (M_SE). Children with SDB show higher SpO_2_ variability reflected M_Δ and a higher number of desaturations M_n2% due to sleep apnea. They also reflect higher power in the modulation band and lower spectral complexity (see Table 3). Quartile values are displayed as bottom, middle and top horizontal line of the boxes. Whiskers are used to represent the most extreme values within 1.5 times the interquartile range from the median. Outliers (data with values beyond the ends of the whiskers) are displayed as circles.

Children with SDB showed a modulated SpO_2_ waveform due to the desaturations caused by OSA. These SpO_2_ fluctuations are reflected in the spectral domain through a clear modulation frequency peak (see Figure 2 for a subject with and without SDB and Figure 3 for the population with and without SDB) and lower spectral complexity or randomness. In children with a high AHI, the time varying PSD of the SpO_2_ illustrated a clear modulation frequency peak relative to children without SDB (Figure 4). The power in the modulation frequency band is positively correlated with the AHI index (r = 0.7, 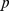-value 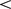 0.0001). Therefore, children with SDB showed higher power in the modulation frequency band and thus a higher power ratio (M_P, M_R), with higher power dispersion overnight (S_P, S_R), and lower spectral entropy (M_SE) than NonSDB children (Figure 6). SDB children also showed higher SpO_2_ variability reflected by M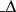, M_iqr, M_std, M_CTM and higher overnight SpO_2_ dispersion associated with S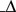, S_iqr, S_std and S_CTM. As expected, the number of desaturations below baseline (M_n2%) and the time spent below 92% (M_t92%) were higher in SDB children (see Tables 3 and 4).

**Table 3:** Effects of SDB on the normally distributed features extracted from SpO_2_ pattern characterization and PRV analysis.

Feature	SDB	NonSDB	Mean diff	95% CI	*p*-value
		SpO_2_ pattern characterization			
**M_R**	0.49 ± 0.04	0.46 ± 0.04	0.03	(0.017, 0.044)	<0.0001
**S_R**	0.14 ± 0.02	0.13 ± 0.01	0.06	(0, 0.01)	0.04
**M_SE**	6.86 ± 0.18	6.98 ± 0.16	−0.12	(−0.18, −0.07)	<0.0001
**S_SE**	0.47 ± 0.09	0.46 ± 0.08	0.02	(−0.01, 0.05)	0.39
**M_Δ**	0.41 ± 0.23	0.26 ± 0.10	0.15	(0.08, 0.21)	<0.0001
**S_Δ**	0.33 ± 0.30	0.18 ± 0.12	0.15	(0.07, 0.24)	<0.0006
**M_iqr**	0.82 ± 0.46	0.51 ± 0.22	0.31	(0.18, 0.44)	<0.0001
**S_iqr**	0.69 ± 0.63	0.40 ± 0.40	0.29	(0.13, 0.46)	<0.0025
**M_std**	0.65 ± 0.33	0.43 ± 0.17	0.22	(0.12, 0.31)	<0.0001
**S_std**	0.52 ± 0.40	0.32 ± 0.25	0.20	(0.1, 0.32)	<0.001
		**HRV characterization**			
**M_LF**	0.34 ± 0.10	0.29 ± 0.10	0.04	(0.01, 0.08)	0.016
**S_LF**	0.15 ± 0.03	0.13 ± 0.05	0.02	(0.01, 0.04)	<0.001
**M_HF**	0.63 ± 0.11	0.68 ± 0.11	−0.05	(−0.09, −0.01)	0.01
**S_HF**	0.15 ± 0.03	0.13 ± 0.05	0.02	(0.01, 0.04)	<0.0001
**M_RR**	0.74 ± 0.14	0.79 ± 0.15	−0.05	(−0.10, −0.002)	0.04
**S_RR**	0.05 ± 0.02	0.05 ± 0.03	0.001	(−0.008, 0.01)	0.82

Two-sample t-tests for unequal variances are applied to the data to obtain 95% confidence intervals (CI) and *p*-values. These features are represented by their mean ± standard deviation for children with and without SDB, their mean difference, CIs, and *p*-value. For simplicity, only the mean (M) and standard deviation (S) of the significant time varying features are represented. For abbreviations see table 2.

**Table 4:** Effects of SDB on the non-normally distributed features extracted from SpO_2_ pattern characterization and PRV analysis.

Feature	SDB	NonSDB	Mean diff	*p*-value_1_	*p*-value_2_
		SpO_2_ pattern characterization			
**M_P**	0.044± 0.07	0.015 ± 0.02	0.03	<0.0001	<0.0001
**S_P**	0.10 ± 0.15	0.05 ± 0.09	0.05	<0.0001	<0.0001
**M_n2%**	0.43 ± 0.41	0.17 ± 0.17	0.26	<0.0001	<0.0001
**S_n2%**	0.76 ± 0.43	0.44 ± 0.23	0.32	<0.0001	<0.0001
**M_t94%**‡	1.64 ± 3.41	1.38 ± 6.48	0.25	<0.0001	<0.0001
**S_t94%**‡	4.70 ± 6.99	2.80 ± 8.45	1.90	<0.0005	<0.0001
**M_CMT**	0.90 ± 0.07	0.94 ± 0.04	−0.04	<0.001	<0.0001
**S_CMT**	0.11 ± 0.05	0.07 ± 0.03	0.03	<0.0001	<0.0001
		**HRV characterization**			
**M_LF/HF**	0.54 ± 0.86	0.22 ± 0.80	0.31	<0.0001	<0.0001
**S_LF/HF**	0.67 ± 0.37	0.46 ± 0.38	0.21	<0.0001	<0.0001
**M_SDNN**	0.07 ± 0.04	0.069 ± 0.07	0.001	0.19	0.08
**S_SDNN**	0.02 ± 0.02	0.04 ± 0.09	−0.02	0.01	0.001
**M_RMSSD**	0.07 ± 0.04	0.08 ± 0.10	−0.01	0.57	0.35
**S_RMSSD**	0.04 ± 0.02	0.05 ± 0.11	−0.01	0.017	0.004

Two-sample t-tests for unequal variances are applied to the log-transformed data to obtain the *p*-values (illustrated by *p*-value_1_). Additionally, Mann-Whitney U tests are also applied to the original data to account for the non-normal data distributions (*p*-value_2_). Features are described by their mean ± standard deviation for children with and without SDB and their mean difference. For abbreviations see table 2. For simplicity, only the mean (M) and standard deviation (S) of the significant time varying features are represented. Two variables, represented by ‡, were transformed using Box-Cox because they contained zero values.

**Figure 7:**
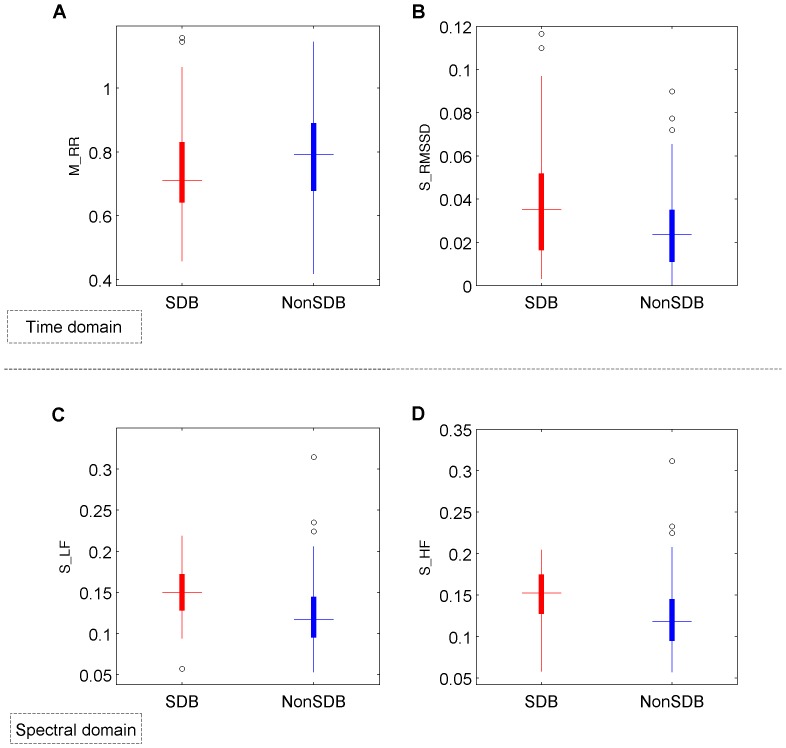
Distribution of PRV features. Boxplot of features extracted from PRV analysis such as (A) the mean of pulse to pulse intervals (M_RR), (B) the standard deviation of RMSSD (S_RMSSD) in time domain, (C) the standard deviation of the normalized power in the LF, and (D) HF band in the spectral domain (S_LF and S_HF, respectively). Children with SDB reflect higher heart rate and PRV dispersion, reflected by a lower pulse to pulse interval and higher standard deviation of the standard PRV measures. Quartile values are displayed as bottom, middle and top horizontal lines of the boxes. Whiskers are used to represent the most extreme values within 1.5 times the interquartile range from the median. Outliers (data with values beyond the ends of the whiskers) are displayed as circles.

With regards to PRV analysis, higher normalized LF power (M_LF), lower HF power (M_HF) and thus higher LF/HF ratio was observed in SDB children, reflecting higher sympathetic activity due to episodes of OSA. They also showed higher heart rate (M_RR) and higher overnight dispersion on PRV measures such as S_LF, S_HF, S_LF/HF, S_SDNN, S_RMSSD (see Tables 3 and 4, and Figure 7).

### 3.2 Feature Selection and Classification

**Figure 8:**
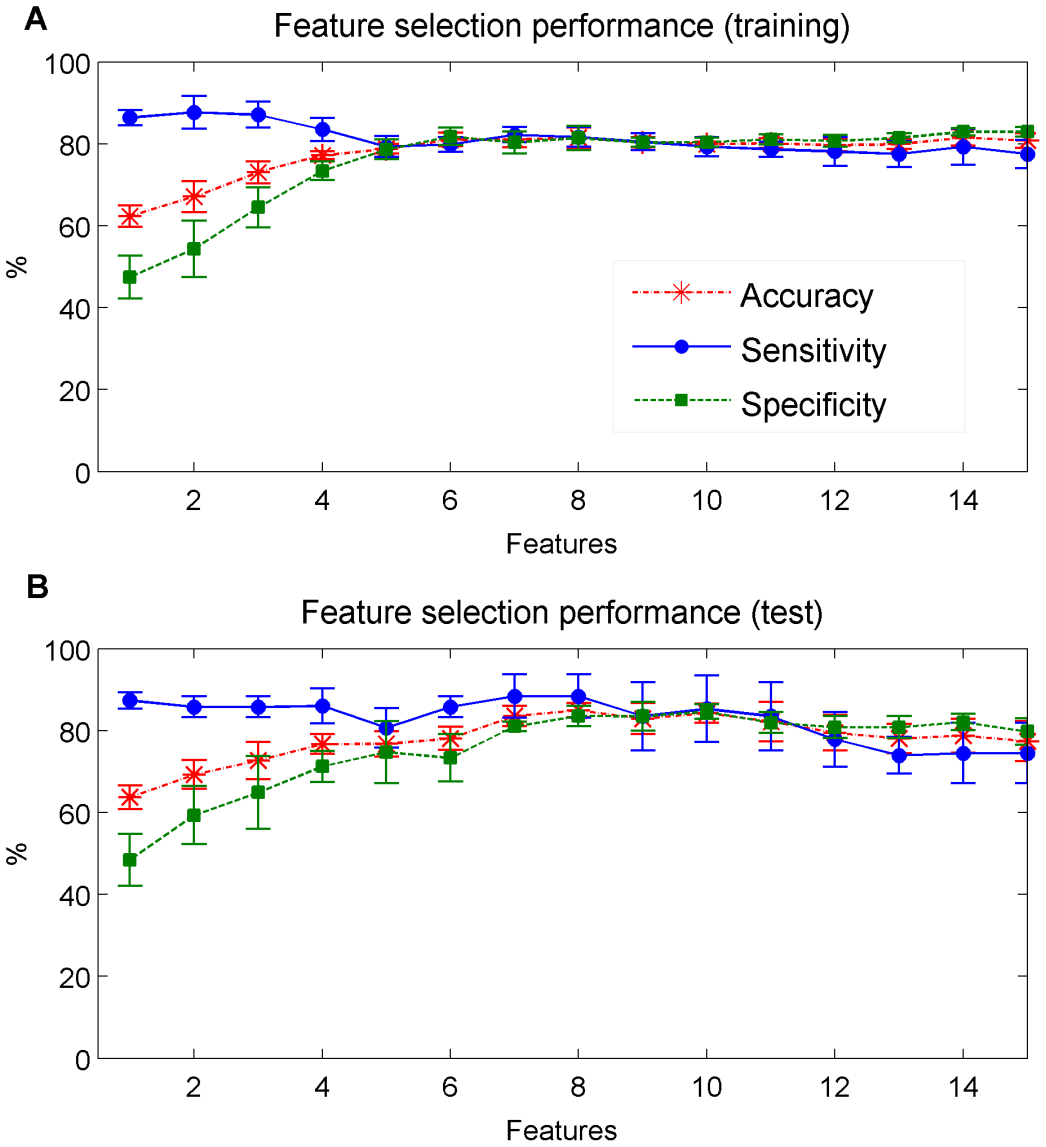
Performance of the feature selection. The performance is represented in terms of accuracy, sensitivity and specificity in classifying SDB and NonSDB children, whenever the feature that provided the higher AUC (with the training set) was included in the linear discriminant. The results obtained with (A) the training dataset (with internal LOO cross-validation) and (B) the test dataset (with external 4-fold cross-validation) are illustrated.

**Figure 9:**
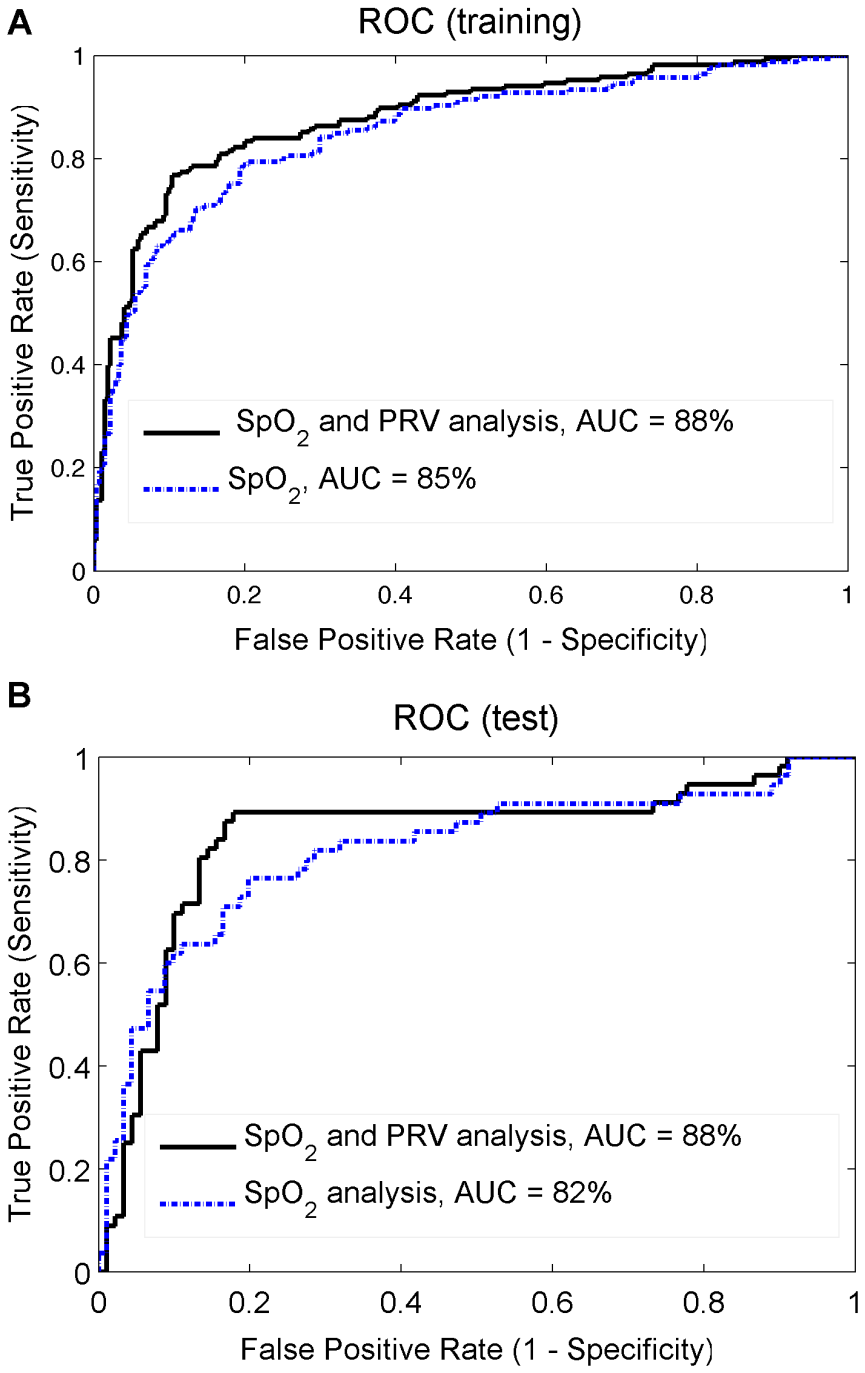
Training and test ROC. The ROC obtained with the 8 most discriminating features (see Table 5) applied to (A) the training dataset (with internal LOO cross-validation) and (B) the test dataset (with external 4-fold cross-validation).

The most discriminating 15 features were selected using the training dataset and evaluated on the test dataset; this process was repeated N = 4 times resulting in 4 external error estimates. The best internally cross-validated AUC = 88% was obtained with 8 features, providing accuracy, sensitivity and specificity rates above 80% for the training dataset (see Figure 8.a and Figure 9.a). The performance of the most discriminating 8 features evaluated on the test dataset (Figure 8.b) provided on average, an AUC of 86%, accuracy of 84.9%, sensitivity of 88.4% and specificity of 83.6%. The positive and negative predictive value showed that only 76.9% of the children classified as SDB would have the disease, and 92.6% of the children classified as NonSDB would not. (See Figure 9.b and Table 5).

**Table 5:** Classification performance based on the linear discriminant analysis using the most discriminatory set of 8 features.

LD performance (Test)	Acc (%)	Sn (%)	Sp (%)	NPV (%)	PPV (%)
**8 features (SpO_2_ and PRV)**	**84.9**	**88.4**	**83.6**	**92.6**	**76.9**
**8 features (SpO_2_)**	78.5	80.0	83.9	87.4	77.6

The results were obtained with the test dataset using 4-fold cross-validation. The performance of combined SpO_2_ and PRV analysis is compared to SpO_2_ analysis alone, in terms of accuracy (Acc), sensitivity (Sn), specificity (Sp) and negative and positive predictive value (NPP and PPV, respectively).

The combined SpO_2_ and PRV analysis improved the performance of the classifier identifying children with SDB. The AUC obtained with the SpO_2_ characterization alone (82%) increased to 88% by including PRV information (see Figure 9).

### 3.3 Performance of the Proposed Feature Set

**Figure 10:**
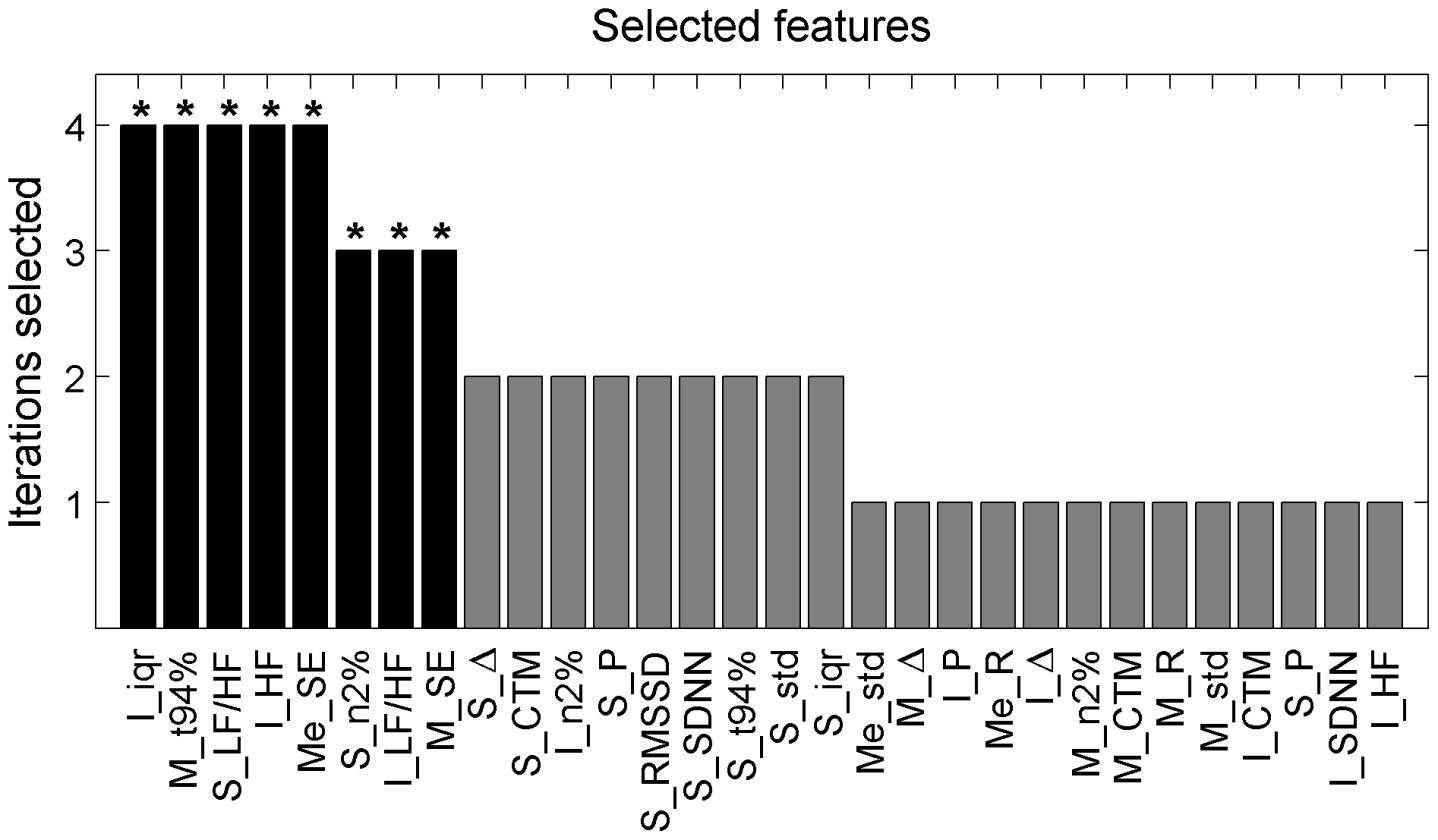
Feature histogram. The histogram of the feature selection process, where the 15 most discriminating features were selected in each iteration. The histogram illustrates the total number of times each feature was automatically chosen by the selection algorithm in each iteration (4 in total). The feature selection was validated internally with a LOO cross-validation and externally with 4-fold cross-validation. The features selected in every iteration (4 times) or nearly every iteration (3 times) were defined as the most discriminating and were proposed as the optimal to create the final linear discriminant. They are represented in black and marked with *.

The feature selection algorithm chose the 15 most discriminating features in each iteration (N = 4 iterations in total). The histogram of the most discriminating features is represented in Figure 10, showing how many times each feature was selected in the four different iterations. 5 features were selected in each of the four iterations and 3 were selected in three of the four iterations, reflecting high discriminant value. Therefore, we selected these 8 features to screen children with and without SDB using a linear discriminant. These most discriminating features (marked with * in Figure 10) are related to the variability and modulation of SpO_2_ and PRV due to intermittent apnea/hypopnea events during the sleep. A linear classifier based on this fixed 8-feature set provided an accuracy of 85.0%, sensitivity of 88.4%, specificity of 83.6%, positive predictive value of 76.0% and negative predictive value of 90.6% using 4-fold cross-validation.

## Discussion

This study shows that combining the SpO_2_ pattern characterization and PRV analysis performed using the Phone Oximeter's measurements (SpO_2_ and PPG), improved the Phone Oximeter's performance as a possible SDB screening tool. In 146 children (SDB prevalence of 38%) the SpO_2_ fluctuations caused by SDB modulated the SpO_2_ signal, which was reflected in the frequency domain by a clear modulation peak and less spectral randomness. Therefore, children with SDB showed significantly greater SpO_2_ variability and overnight dispersion in the time domain, accompanied by higher SpO_2_ spectral power at low frequencies (modulation band) and lower spectral complexity in the frequency domain, than NonSDB children.

SDB children showed higher sympathetic activity as a response to intermittent hypoxia and arousals during sleep. This was reflected by a significantly higher normalized power at low frequency and lower normalized power at high frequency, resulting in a higher LF/HF ratio. These results confirmed previous findings about cardiac modulation in subjects with SDB [19],[20],[37],[38].

The most discriminating features identifying children with SDB were automatically selected (with internal cross-validation) and evaluated (with external cross-validation). The selected features were related mainly to the spectral analysis of PRV, and SpO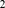 variability and modulation represented in the spectral domain. This reflects the significant effect of intermittent apnea events and respiratory arousals in the sympathetic and parasympathetic activity, and the recurrent desaturations in the SpO_2_ pattern variability. The best performance, obtained with 8 features, provided higher accuracy, sensitivity and specificity values than the SpO_2_ pattern characterization alone. The results showed that when using the Phone Oximeter as an SDB screening tool, 88.4% of the children with SDB would be correctly identified. However, 23.1% of the children misclassified as having SDB, would be unnecessarily sent for a PSG, and 7.4% of the children with SDB, would be wrongly classified as NonSDB and remain undiagnosed. Based on the feature selection histogram, a fixed set of the most frequently selected features was suggested to create the optimal linear discriminant. Similar cross-validated classification results were obtained with the proposed optimal linear discriminant.

Our results, obtained with the Phone Oximeter, are comparable with previous studies with more sophisticated approaches or devices. Heneghan et al. proposed a combined Holter-Oximeter as a portable home-based device to automatically assess OSA in adults with signs of SDB [19],[20]. Their system provided an automatic epoch-by-epoch estimate of OSA occurrence and calculated an AHI for each subject. Overall the system correctly identified 85.3% of all 1-minute epochs. Chung et al. reported that oxygen desaturation index (ODI), calculated from nocturnal oximetry, was a good predictor of AHI in adult surgical patients [39]. An ODI 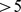 provided an accuracy of 87%, sensitivity of 96.3% and specificity of 67.3% identifying adults with an AHI 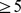. In this study, we focused on identifying children with SDB, which is more challenging than in their adult counterparts. Yet, the Phone Oximeter alone provided similar accuracies, maintaining a good sensitivity-specificity balance.

Gil et al. successfully associated amplitude fluctuations in the PPG signal with SDB, and used HRV calculated from ECG to discriminate between amplitude fluctuations related or unrelated to apneic events [37], [38]. In a similar study, they recently proposed using pulse rate variability (PRV) instead of HRV [40] and reported an accuracy of 86.67% in identifying children with SDB. However, their dataset consisted of 21 children, 10 of whom were diagnosed with SDB. In our previous study, we obtained similar results (accuracy 86.8%) characterizing the SpO_2_ pattern of 68 children, 30 of whom were diagnosed with SDB [24]. In this study, with a bigger cohort (146 children), we showed that combining SpO_2_ pattern characterization [24] and PRV analysis [28], the Phone Oximeter provides a more robust stand-alone approach to screening for SDB in children. The sensitivity increased from 80% to 88%, reflecting that with this analysis we were able to detect more OSA events (perhaps those that occur in absence of SpO_2_ desaturation). Furthermore, compared to ECG recordings, PPG recordings are more convenient to obtain, with the potential of being used at home. Nixon et al. successfully proposed and validated a score system based on overnight oximetry to prioritize surgery [12], [13]. The aim of our study is instead to provide a screening tool that would prioritize children for referral to a formal sleep laboratory, such as at BCCH, for PSG. We do not intend to diagnose SDB with the Phone Oximeter, but to have an intermediate at-home monitoring step that will reach more children suspected of having SDB in a timely and less-stressful means.

**Figure 11:**
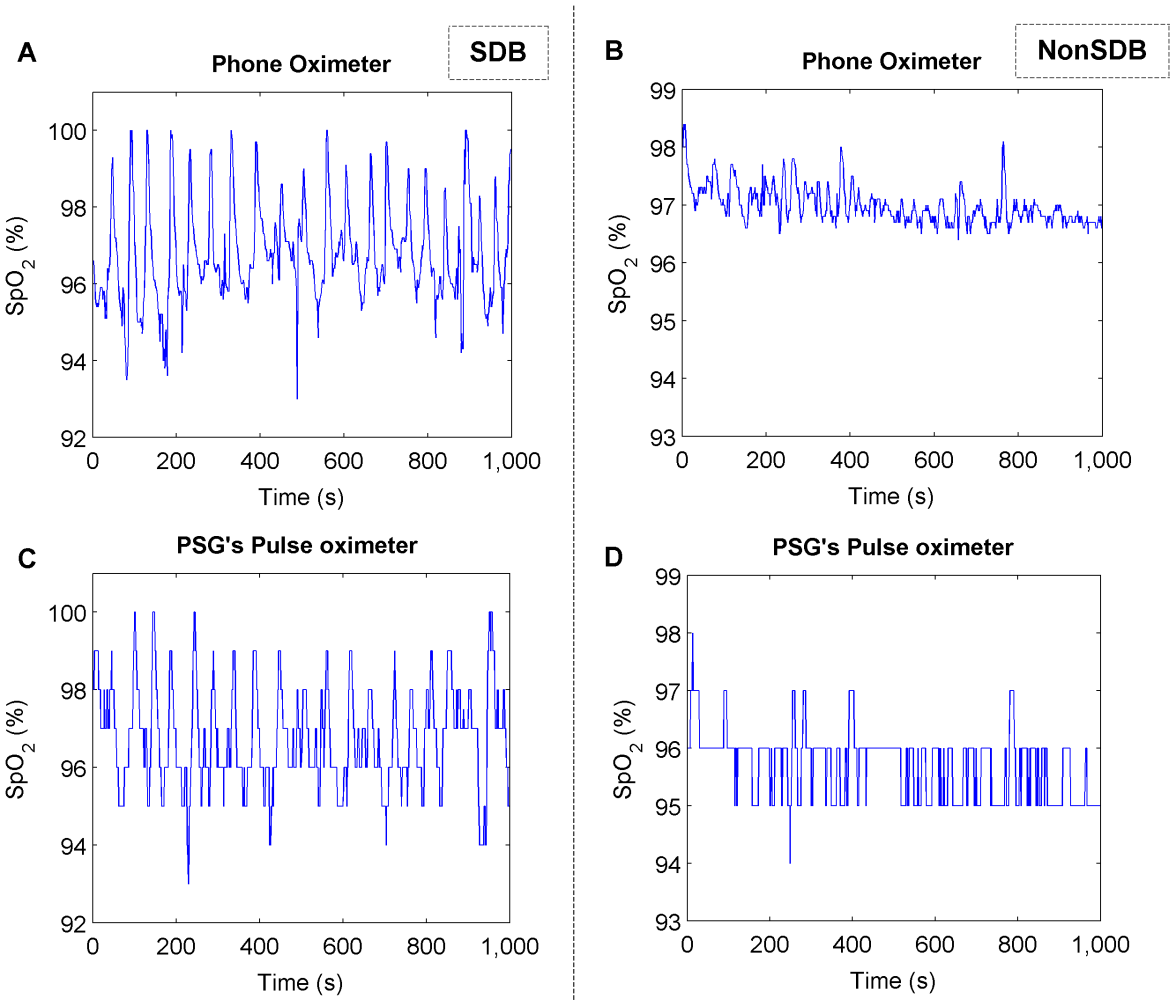
SpO_2_ signal with different resolutions. An SpO_2_ signal segment, recorded using the Phone Oximeter (0.1% resolution) for (A) SDB and (B) NonSDB children, and the corresponding SpO_2_ signal recorded simultaneously with the PSG's pulse oximeter (1% resolution) for the same SDB (C) and NonSDB (D) children. The SpO_2_ resolution has a great influence in regularity measures like approximate entropy and Lempel-Ziv [41]. Therefore, SpO_2_ resolution should be taken into account when studying the SpO_2_ pattern in children with SDB.The SpO_2_ randomness shown for NonSDB children in 0.1% resolution SpO_2_ signal (provided by the Phone Oximeter), is not reflected in the 1% resolution SpO_2_ signal (provided by the PSG's pulse oximeter) because of the rounding effect. This resolution difference might be the reason why children with SDB showed a higher complexity than NonSDB children with conventional pulse oximeter.

Álvarez et al. proposed SpO_2_ regularity as useful information to improve SBD diagnosis in adults and showed that subjects with SDB had lower SpO_2_ regularity than NonSDB subjects [15]. However, we obtained no significant difference in the SpO_2_ regularity between SDB and NonSDB children. In a recent study, we found that theSpO_2_ resolution had a major influence on regularity measurements and demonstrated that different resolutions provided different results. As it is illustrated in Figure 11, higher SpO_2_ resolution permits observation of the small changes in the SpO_2_ signal, which have a great impact on the complexity value of the SpO_2_ signal. Therefore, the devices' resolution should be carefully considered when dealing with SpO_2_ regularity to identify children with SDB [41].

Considering the population in British Columbia under 14 years old (16% of 4,609,946 [42]), in conjunction with SDB prevalence [2] of 2%, around 14,750 children would suffer from SDB. In this study, 38% of children with signs of SDB referred to BCCH for a PSG, were diagnosed with SDB upon analysis of a full PSG. Therefore, approximately 38,815 children with signs of SDB may require a PSG at BCCH, where only 250 PSGs can be performed per year. The availability of PSG does not meet the demand requirements, and results in long waitlists. The results of this study show that using the Phone Oximeter as a screening tool prior to PSG could reduce the number of PSGs required to less than half, while effectively studying the same number of children. From the 56 SDB children studied, 50 would have been correctly screened and sent for a PSG, while 6 cases of SDB would have remained undetected in the first screening test (false negatives). From the 90 NonSDB children studied, 75 would have been correctly classified, while 15 children (false positives) would have been sent for PSG unnecessarily. In total, only 65 out of the 146 children (44%) would have been referred to the hospital for a PSG. With a capacity of only 250 PSGs per year at BCCH, and considering that only 44% of the screened children require a full PSG, we can back-calculate that the number of patients that it is possible to screen using the Phone Oximeter under current hospital limitations is 568 per year (250 would be sent for a PSG, while the remaining 318 children, would be watched for progression of symptoms). In this manner, more than double the number children with signs of SDB could be screened each year, and sent for PSG if required. Therefore, using the Phone Oximeter would result in increased coverage of medical services to children in British Columbia with signs of SDB, reducing wait times and optimizing usage of hospital resources.

The Phone Oximeter provides the perfect platform to create an SDB screening prototype, permitting overnight pulse oximetry recordings and allowing implementation of the algorithm on a smartphone. In addition, it can wirelessly communicate information (raw data, results etc.). More sophisticated analysis approaches such as the correntropy spectral density [25], [43], could be applied to the SpO_2_ for a more robust spectral analysis that includes nonlinear information. However, simpler algorithms are preferred so that they can be easily implemented on a smartphone with low computational load. By using the low cost version of the Phone Oximeter, which interfaces the sensor directly with the phone via the audio jack, the cost to monitor SDB with the phone will be reduced to that of the finger probe alone. The offline SpO_2_ and PRV analysis for the overnight study of each subject takes between 1 to 2 seconds. Real time performance is not required, since we aim to provide a final screening result after the overnight recording.

## Limitations of the Study, Further Questions, and Future Work

The pediatric population of this study includes children with a higher likelihood of SDB than the general population, having already been referred to BCCH for a PSG. Although our target population for the SDB screening tool is children with signs of SDB, the utility of the Phone Oximeter in a general population with a lower prevalence of SDB is presently unproven.

A limitation of this study is that the recordings were performed in a hospital sleep laboratory at the BCCH. At-home screening is our goal for the next study. During recordings performed at home, we expect artifacts caused by sensor displacement to be more severe, which could degrade the performance of the Phone Oximeter as an SDB screening tool. Therefore, the implementation of an accurate artifact detection technique for the PPG and SpO_2_ signals, directly on the phone, is one of our main future challenges.

Previous studies suggest that the indication for SDB treatment, primarily adenotonsillectomy, is an AHI (from PSG)>5, which coincides with the current practice at BCCH. Therefore, in this study we considered children with an AHI 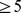 as positive for SDB. However, there is no discrete definition of OSA based on AHI alone, but rather a continuum from normal to abnormal. We recognize that some studies consider an AHI 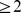 as abnormal or mild OSA. For example, The Childhood Adenotonsillectomy Trial (CHAT), designed to evaluate the efficacy of early adenotonsillectomy versus watchful waiting with supportive care, defined OSA as an AHI score 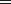 2. Surgical treatment did not significantly improve attention or executive function in these patients, but did reduce OSA symptoms. However, the population in the CHAT study primarily had mild cases of OSA, reflected by the AHI interquartile range (2.5 to 8.9) in the OSA positive group, which may have affected their assessment of treatment efficacy. Therefore, we will further investigate the Phone Oximeter's performance identifying children with SDB based on different AHI thresholds (AHI 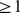, AHI 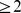), using different classifiers. An AHI 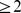 will result in a recommendation for at-home monitoring, and an AHI 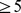 will result in a referral to BCCH for a PSG.

The most common cause of SDB in children is adenotonsillar hypertrophy [4], [5]. Most children can be discharged the same day following adenotonsillectomy surgery, however, those with advanced SDB have a 20-fold higher risk of post anesthetic respiratory complications. Therefore, the aim of our follow-up study is to adapt and test the innovative SDB screening tool in children with suspected SDB before adenotonsillectomy and the incidence of desaturation or ongoing SDB in the days following surgery [8],[44].

## Conclusion

The time-varying characterization of the SpO_2_ pattern and PRV is a suitable tool to provide further knowledge of SpO_2_ and cardiac modulation during sleep apnea, to identify children with SDB. This provides the potential for the Phone Oximeter to be used as an SDB screening tool, providing a portable at-home device with the capability of monitoring patients over multiple nights. At-home screening will result in less sleep disturbance, facilitate a more natural sleep pattern and prevent unnecessary burden to both families and the health care system. Additionally, this tool has the potential to optimize resources by identifying those children who should undergo a complete PSG test.
